# Early essential newborn care in national tertiary hospitals in Cambodia and Lao People’s Democratic Republic: a cross-sectional study

**DOI:** 10.1186/s12884-022-05056-5

**Published:** 2022-10-04

**Authors:** Tomomi Kitamura, Hiromi Obara, Mari Honda, Tomoko Mori, Tomoo Ito, Mari Nagai, Sommana Rattana, Tung Rathavy, Yasuo Sugiura

**Affiliations:** 1 Bureau of International Cooperation, National Center for Global Health and Medicine, 1-21-1, Toyama, Shinjuku Tokyo 1628655 Japan; 2 Department of Pediatrics and Adolescent Medicine, Tokyo Medical University Hospital, 6-7-1 Nishishinjuku, Tokyo, Shinjuku-ku 160-0023 Japan; 3 Ministry of Health, XJ48+FFP, Ban thatkhao, Sisattanack District, Rue Simeuang, Vientiane, Laos; 4 University of Health Sciences, Phnom Penh, Cambodia

**Keywords:** Essential newborn care, Newborn health, Quality of care

## Abstract

**Background:**

Ministries of health in collaboration with the World Health Organization Regional Office for the Western Pacific (WPRO) have been scaling up early essential newborn care (EENC). This study was carried out to understand current EENC practices at hospitals in two priority countries: the Kingdom of Cambodia (Cambodia) and Lao People’s Democratic Republic (Lao PDR).

**Methods:**

EENC is subdivided into 79 checkpoints, referencing the self-monitoring checklist developed by the WPRO. Each checkpoint is rated using a 0 to 2-point scale, and a percentage was calculated for the rate of practice of each checkpoint by dividing the total scores by the maximum possible scores.

**Results:**

In total, 55 and 56 deliveries were observed in Cambodia and Lao PDR, respectively, and 35 and 34 normal deliveries were included in the analysis. The overall rates of the practices within the first 15 minutes after birth were high in both countries. The rates of the practices before birth and 15 minutes after birth were lower than the rates of the practices performed within the first 15 minutes after birth, especially “hand wash before preparation”, “preparation for newborn resuscitation”, and “monitoring of postpartum mothers and babies”. A detailed analysis revealed that the quality of the practices differed between the two countries regarding skin-to-skin contact and breastfeeding support.

**Conclusions:**

The high rates of the practices within the first 15 minutes after birth suggest that the EENC coaching sessions supported by ministries of health and the WPRO have been effective. Differences in the quality of practices performed at a high rate between the two countries appeared to be related to factors such as the timing of the study, the perception of the staff, and the situation at the health facilities. These differences and identified practices with lower rates should be improved according to the situation in each country or health facility. Therefore, determining the quality of the practices in a country or a health facility is important. To further improve the quality of EENC, interventions tailored to the specific situation are necessary.

## Background

Despite a substantial reduction in the number of childhood deaths since 1990, the number of deaths during the newborn period has been decreasing at a slower rate [1]. In 2014, Every Woman Every Child together with the World Health Organization (WHO) and the United Nations Children’s Fund (UNICEF) developed “Every Newborn: An Action Plan to End Preventable Deaths” in response to a demand by countries to set out a clear vision of how to improve newborn health and prevent stillbirths by 2035 [2].

The WHO Regional Office for the Western Pacific (WPRO), together with UNICEF East Asia and Pacific regions, ministries of health, academics, and nongovernmental organisations in the regions, developed “The Action Plan for Healthy Newborn Infants in the Western Pacific Region (2014-2020)” in 2014 [3]. It focuses on improving the quality of early essential newborn care (EENC), and its goal is to eliminate preventable newborn mortality by providing universal access to high-quality EENC [3].

EENC includes the care provided during and immediately after birth to all mothers and newborn infants and also the prevention and care for preterm and low-birth-weight infants [3]. The core of EENC is “The First Embrace” which consists of “immediate drying”, “immediate skin-to-skin contact”, “appropriately timed clamping and cutting of the cord”, and “promotion of exclusive breastfeeding” [3].

Eight countries with high neonatal mortality in the Western Pacific region were selected as priority countries to implement the action plan with support from the WHO WPRO [4]. After an initial coaching session on EENC, each health facility set up its hospital EENC team. The quality of EENC was closely assessed and improved by the team using a module called “Introducing and Sustaining EENC in Hospitals: Routine Childbirth and Newborn Care” which was developed by the WHO WPRO. In addition, nationwide annual reviews were conducted for selected health facilities [5, 6]. The purpose of this study was to observe EENC practices at hospitals in two of the eight priority countries: the Kingdom of Cambodia (Cambodia) and Lao People’s Democratic Republic (Lao PDR). Moreover, we aimed to shed light on the quality of and adherence to EENC using the same modules as those used by the WHO WPRO but with external observers to address the “know-do” gap in detail since it is important to document these to further improve the programme outcomes.

## Methods

### Study countries and health facilities

Two of the priority countries, Cambodia and Lao PDR, were purposefully selected due to the presence of research centres in collaboration with the National Center for Global Health and Medicine in Japan. The study was conducted in Lao PDR from January to February 2016 and in Cambodia in September 2016. Four national hospitals in the capital of Lao PDR and two national hospitals in the capital and one provincial hospital in Cambodia were selected as target hospitals since these hospitals had previously received the initial coaching by the WHO WPRO. Moreover, they were considered to be key hospitals to disseminate EENC nationwide. The two countries differed in when and how EENC had been implemented (Table 1). In Cambodia, EENC was initiated in 2011, and coverage was expanded to primary health facilities in 2016. All the target hospitals in Cambodia had been practising EENC for more than 4 years when the study was conducted. In Lao PDR, EENC was initiated in 2015, and the staff at the target hospitals were newly trained when the study team visited.

**Table 1:** The number of health facilities where EENC has been implementation

	2015	2017
Cambodia	1136 (91%) < 1246>^a^	1139 (90%) < 1272>^a^
Lao PDR	23 (2%) < 1145>^a^	53 (18%) < 299>^a^

^a^total number of delivery centres

### Study design

This was a cross-sectional observational study, and a team of external observers conducted assessments of EENC clinical practices using the checklists in the module “Introducing and Sustaining EENC in Hospitals: Routine Childbirth and Newborn Care” [5]. The study team consisted of four Japanese paediatricians who were trained in general paediatrics and neonatology and were familiar with EENC. One or two team members stayed in the delivery rooms of the target hospitals during the daytime and overnight in one national hospital in the capital of Cambodia. The checklists were modified, and several observation items were added according to the research purpose; however, the contents and intentions of the original checklists were respected and not altered. Observations started after a pregnant woman entered the delivery room and the staff confirmed her cervix was fully dilated, or they started to prepare the patient for the delivery. Observations lasted until 90 minutes after the birth of the baby but were discontinued if the mother or baby needed special medical attention or treatment such as an emergency caesarean section or newborn resuscitation.

### Study population and cases

Healthcare workers involved in uncomplicated spontaneous deliveries in the target hospitals during the study period were observed when the researchers were present. They are skilled birth attendants including midwives, nurses and medical doctors. All caesarean sections and deliveries of newborn babies who presented with acute or chronic conditions which required special medical attention were excluded from the observations.

### Data collection

Clinical practices regarding EENC were observed every day from 8:00 to 17:00, except for Saturdays and Sundays, during the study period using the standard checklist mentioned above. Night observations started at 17:00 and ended at 8:00 the next day.

### Data analyses

EENC is subdivided into 79 practices referenced in the monitoring checklist developed by the WPRO. Each practice is rated using a 0 to 2-point scale (fully practised: 2 points, partially practised: 1 point, not practised: 0 points). The practice was assessed as “partially practiced” when a health care worker missed any component of the practice in the checklist. A percentage was calculated for each practice by dividing the total scores by the maximum possible scores to generate a “practice rate” for descriptive analysis.

### Ethical approval and consent to participate

This study was conducted after receiving ethical approvals from National Ethics Committee for Health Research, Cambodia (reference number: 255NECHR, obtained on 27/6/2016), National Institute of Public Health, Lao PDR (reference number: 065/2015 NIOPH/NECHR, obtained on 3/11/2015), and the Institutional Review Board of the National Center for Global Health and Medicine, Japan (reference number: NCGM-G-001849-00, obtained on 11/12/2015). Written informed consent was obtained from all the healthcare workers who were involved in the uncomplicated spontaneous deliveries during the observation periods in the target hospitals. All the methods were carried out in accordance with relevant guideline and regulation including the Declaration of Helsinki.

## Results

**Fig. 1:**
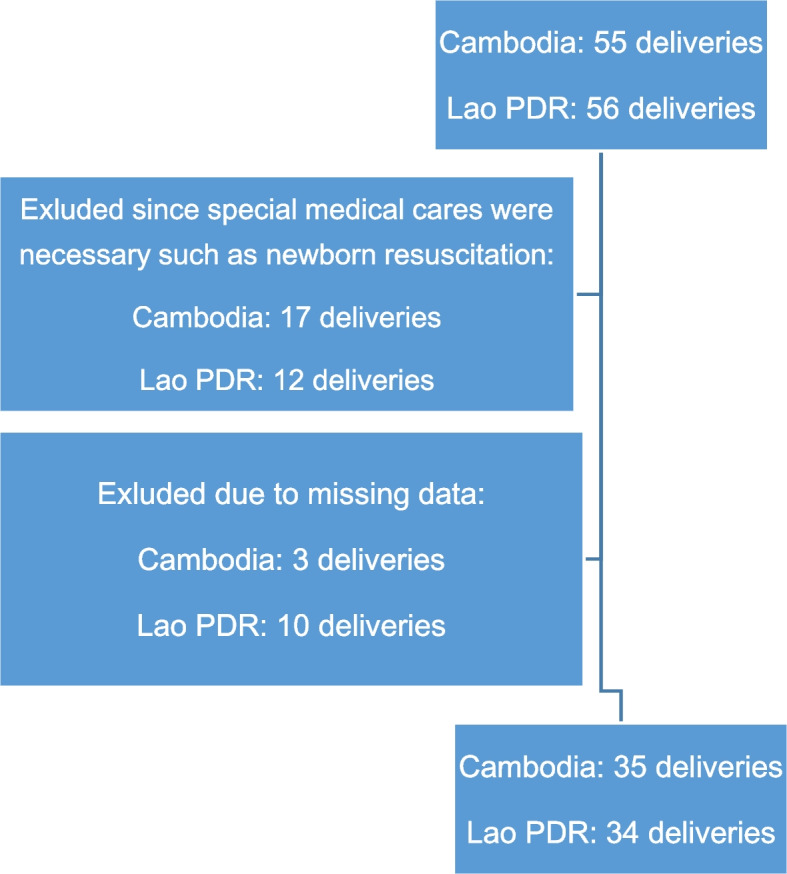
Study profile. Time to start SSC: missing 2 (Cambodia). Time to give oxytocin: missing 1 (Cambodia), 1 (Lao PDR). Time to cord clamping: missing 2 (Lao PDR). Time to leaving delivery room: missing 1 (Lao PDR)

A total of 111 deliveries were observed (Cambodia: 55, Lao PDR: 56). Twenty-nine deliveries were excluded since special medical care was required for either the mother or her newborn baby (Cambodia: 17, Lao PDR: 12). Thirteen deliveries were excluded due to missing data (Cambodia: 3, Lao PDR: 10). Finally, 69 deliveries were included in the analysis (Cambodia: 35, Lao PDR: 34) (Fig. 1).

**Fig. 2:**
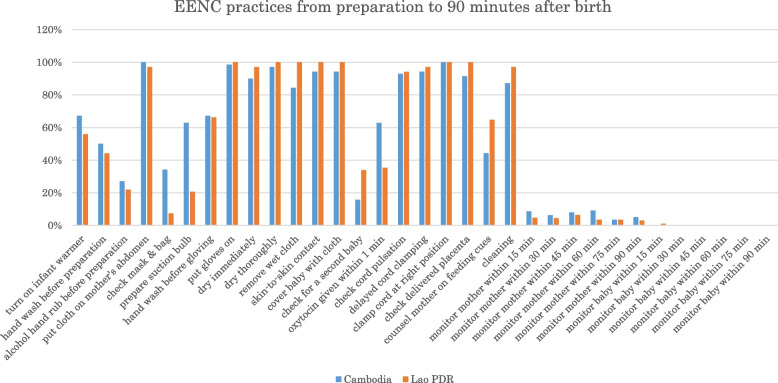
EENC practices from preparation to 90 minutes after birth

Both countries showed a similar pattern of practice rates throughout the course of the delivery (Fig. 2).

The practice rates within the first 15 minutes after birth were more than 80% in both countries, except for “checking for a second baby before giving oxytocin” (16% in Cambodia, 34% in Lao PDR) and “giving oxytocin within 1 minute of birth” (63% in Cambodia, 35% in Lao PDR). Three practices included in “The First Embrace”, “immediate drying” (90% in Cambodia, 97% in Lao PDR), “immediate skin-to-skin contact” (94% in Cambodia, 100% in Lao PDR), and “appropriately timed clamping and cutting of the cord” (94% in Cambodia, 97% in Lao PDR), were provided at a rate of more than 80% in both countries.

The rates of the practices performed before birth and 15 minutes after birth were lower than the rates of those performed within the first 15 minutes after birth, especially practices related to hand hygiene, preparation of newborn resuscitation, and monitoring of postpartum mothers and babies. Both countries demonstrated very high practice rates for the use of surgical gloves (99% in Cambodia, 100% in Lao PDR); however, the practice rates of proper handwashing before preparation for the delivery was 50% in Cambodia and 44% in Lao PDR and hand washing before gloving was 67 and 66%, respectively. The study team observed that handwashing was not carried out, especially during emergencies, and that the staff sometimes touched unclean surfaces (e.g., mobile phones) after handwashing. The practice rate of turning on the infant warmer (or turning on any warming device if the proper infant warmer was not available) was 67% in Cambodia and 56% in Lao PDR. The practice rates of preparing and testing the ventilation bag and mask were 34% in Cambodia and 7% in Lao PDR. The practice rates of preparing the suction device were 63 and 21% in Cambodia and Lao PDR, respectively. Formal monitoring of mothers and babies after delivery for any of the vital signs (five vital signs for mothers and three vital signs for babies) occurred at a rate of less than 10% in both countries.

### Skin-to-skin contact (SSC)

**Fig. 3:**
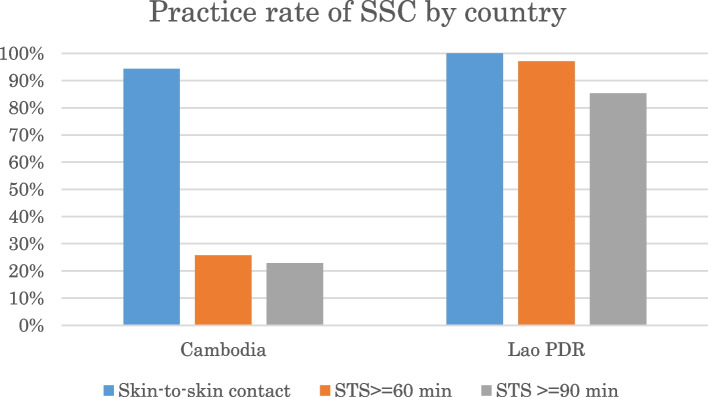
Practice rate of SSC by country

The practice rates of immediate SSC were comparably high in both countries. Figure 3 shows that the practice rates of uninterrupted SSC at 60 minutes and 90 minutes after birth in both countries were 26 and 23%, respectively, in Cambodia and 97 and 85%, respectively, in Lao PDR.

The average duration of uninterrupted SSC was 34 minutes and 87 minutes in Cambodia and Lao PDR, respectively. SSC was initiated immediately after birth; however, it was interrupted to perform routine care such as weighing or administering Vitamin K to the baby. The baby was normally placed back onto the mother’s chest after the routine care was completed.

### Breastfeeding support

**Fig. 4:**
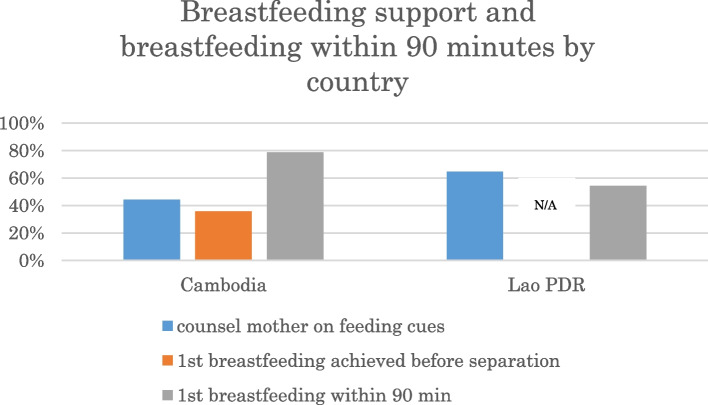
counseling of mothers on feeding cues and first breastfeeding within 90 minutes after birth

The practice rate of “counsel a mother on feeding cues” was 44% in Cambodia and 65% in Lao PDR (Fig. 4).

In Cambodia, 36% of babies achieved first breastfeeding before separation, and 79% of babies breastfed within 90 minutes after birth. In Lao PDR, 54% of babies spontaneously breastfed within 90 minutes of birth. In Lao PDR, first breastfeeding achieved before separation was not recorded since over 80% of babies were not separated from their mothers until 90 minutes after birth. Hands-on breastfeeding support was observed more often in Cambodia, especially after the baby was returned to the mother following routine care.

## Discussion

This study showed the rates at which the EENC practices were performed at national tertiary hospitals in Cambodia and Lao PDR after EENC coaching/training. Both countries demonstrated a similar pattern of practice rates throughout the course of delivery, despite differing situations regarding the implementation of EENC. The rates were high for practices performed within the first 15 minutes after birth; however, it was lower for practices performed before birth and 15 minutes after birth. Moreover, the quality of practices differed slightly regarding SSC and breastfeeding practices.

Previous studies from India showed significant improvements in a number of essential childbirth-related practices and higher adherence in facilities using the coaching-based WHO Safe Childbirth Checklist program [7–11]. For example, a pilot pre-post-intervention study in a sub-district level birth centre in Karnataka, India showed that delivery of essential practices increased from an average of 9.8 (95% confidence interval [CI] 9.4, 10.1) of 29 practices at baseline to an average of 25 (95% CI 24.6, 25.3) [7]. Observations were conducted right after the intervention implementation, and the practice rates, which were calculated as the completion rates of each practice over all the observed deliveries, were high throughout the course of the delivery, except for “partograph use” (42.6%), “appropriate maternal infection management” (75.6%), “intrapartum counselling” (78.3%), “oxytocin given within 1 minute” (68.9%), and “appropriate newborn infection management (within 1 hour of birth)” (31.1%) [7]. Both provider behaviour and health systems need to be modified to improve the quality of care practices. Furthermore, evidence has shown that supportive supervision, clinical mentorship, and coaching can be more effective to change provider behaviour in a variety of settings, increase the rate of skill transfer or adoption, and generate more sustained improvement in performance than training alone [12]. Thus, coaching/training was considered to be one of the methods to facilitate behaviour changes and achieve high practice rates of basic childbirth care to close the “know-do” gap. Therefore, the results of the present study are compatible with the results of the Indian studies [13]. The studies from India demonstrated that it required 1) leadership engagement and commitment, 2) focused introduction of the self-monitoring tools, such as checklists, to end-users, 3) support through coaching to build respectful relationships and communication, and 4) ongoing monitoring and feedback on intervention uptake, problem-solving, and behaviour changes to improve quality of care [8, 13]. Further research is necessary to discover which components of coaching are most effective at facilitating behaviour changes and other systemic changes, and in which contexts these changes take place [13].

Previously and in the present study, some practices have been shown to be resistant to change. Two studies in Uttar Pradesh, India showed similar patterns of practices rates to the present study throughout the course of the delivery, even though they observed and recorded different and fewer practices [8, 9]. Observations were conducted 4 to 12 weeks after the intervention in the study with an adaptive study design prior to a large-scale randomised controlled trial; the practice rates remained lower than 80% for “proper handwashing (21%)”, “baby temperature taken (64%)”, and “breastfeeding within 1 hour of delivery (64%)” even though handwashing and breastfeeding showed significant improvements [8]. Observations were conducted after 2 and 12 months for the matched-pair, cluster-randomised, controlled trial; measuring maternal vital signs (blood pressure/temperature), “hand hygiene (35.3% [2 months] and 12.4% [12 months])”, “no oxytocin given before delivery (67.8% [2 months] and 51.8% [12 months])”, “oxytocin administered within 1 minute after delivery (79.5% [2 months] and 53.9% [12 months])”, “newborn temperature taken (43.0% [2 months] and 22.5% [12 months])”, “skin-to-skin contact initiated at birth (78.9% [2 months] and 68.5% [12 months])”, “skin-to-skin contact maintained for 1 hour (19.3% [2 months] and 5.1% [12 months])”, and “initiation of breastfeeding (69.6% [2 months] and 36.9% [12 months])” remained lower than 80% at the intervention sites [9]. These practices showed significant improvements compared to the control sites both after 2 months and 12 months, except for “no oxytocin given before delivery” and “skin-to-skin contact maintained for 1 hour” which had no data after 12 months [9]. Another study from Uttar Pradesh, India compared adherence to practices with or without the presence of healthcare workers who worked directly with birth attendants (coaches) at 2 months after the implementation of the interventions [11]. The practices which showed moderate and major significant differences without the presence of healthcare workers were “oxytocin administration within 1 minute of birth”, “breastfeeding within 1 hour of birth”, “hand hygiene”, “measuring the baby’s temperature”, and “measuring mother’s blood pressure and temperature” [11]. Hirschhorn et al. speculated that the reason behind this resistance may be that healthcare workers were unable to see the immediate benefits of the practices, and Delaney et al. also pointed out that the practices with early, clear, and definite benefits were more likely to be incorporated into routine practices and lead to behaviour changes [8, 11].

As shown by the present study, routine practices such as hand hygiene and monitoring of postpartum mothers and babies could easily be overlooked even with effective coaching. The present study also showed that preparation for neonatal resuscitation could be overlooked.

Furthermore, this study showed that both countries demonstrated very high practice rates for the use of surgical gloves. However, the practice rates of proper handwashing were lower; therefore, raising awareness of hand hygiene before any procedure is necessary since healthcare-associated infection is a major problem in both developed and developing countries. Moreover, many studies have demonstrated the effect of hand hygiene on reducing those infections [14]. Staff should be aware that gloving does not modify hand hygiene indications or replace handwashing with soap and water or hand rubbing with an alcohol-based hand rub [14]. The use of an alcohol-based hand rub (locally produced/WHO-recommended formulation) may increase compliance with hand hygiene in urgent cases when there is no time for proper handwashing and the hands are visibly clean [14]. Hand hygiene after handwashing also has to be emphasised. EENC coaching/training contains a hand hygiene awareness section, and maintaining awareness in practice is essential.

The EENC clinical pocket guide suggests that the mother and baby should not be left alone and that breathing and temperature should be monitored every 15 minutes [15]. Health facilities or ministries of health have their own guidelines or standards for monitoring mothers; these guidelines normally suggest monitoring of blood pressure and bleeding of postpartum mothers; however, guidelines on the monitoring of babies are not clear. Although the transition from foetal to neonatal life is one of the most vulnerable periods, no hazard outcomes were observed during the study period, and continuous monitoring is necessary to prevent the occurrence of a tragedy [16, 17].

The EENC coaching guideline “Coaching Guide for the First Embrace: Facilitator’s Guide (Early Essential Newborn Care)” recommends setting up a newborn resuscitation area for all deliveries since it is impossible to predict which babies will need resuscitation [18]. The guideline also recommends that an infant warmer should be turned on, if available, and that cloth, a ventilation bag, and a mask should be prepared, a suction device, hat, and clock/timer should be in easy reach/view of the newborn resuscitation area, and that all equipment should be tested before delivery [18]. Although healthcare workers prepared the bag and mask, the devices were not tested frequently. A casual interview in Lao PDR revealed that resuscitation was normally considered to be the responsibility of the paediatricians, which partially explained the relatively low rate of preparation of the bag and mask by the delivery room staff. Preparation of the suction device appeared to be problematic since routine suctioning of newborn babies is no longer recommended; however, the staff were tempted to perform suctioning if the device was readily available [18].

Upon close examination, the present study demonstrated that the quality of EENC practices differed between the two countries regarding SSC and breastfeeding; similar results were also observed in the previous studies from India [9, 11]. The average duration of SSC in Cambodia was 30 minutes since the babies were taken away from the mothers for routine care such as weighing. Subsequently, the babies were returned to the mothers to resume SSC. SSC in Lao PDR was normally uninterrupted for 90 minutes. It is not possible to determine determinants of this difference between two countries due to a nature of the study, however, it could be because the study took place shortly after the training in Laos (after 1 year) comparing to Cambodia (after 4 years) or the coaching focused more on initiation of SSC rather than uninterrupted SSC in Cambodia. Semrau et al. pointed out that only 19% of mother-and-infant pairs maintained SSC for 1 hour when observed 2 months after the implementation of coaching [9]. The benefits of immediate/early SSC, including optimal thermal control together with drying and delayed bathing, higher stability of the cardio-respiratory system (SCRIP) scores, a significant increase in breastfeeding at 1 to 4 months of age, and increased duration of breastfeeding, have been investigated by numerous studies [1, 16, 19]. A Cochrane review with high heterogeneity showed that babies who experienced SSC tended to breastfeed successfully during their first feed [16]. In addition, sustained SSC initiates colonisation with maternal flora, olfactory learning, successful intake of colostrum, and sustained breastfeeding [16, 20]. A clear dose-response effect or the optimal duration of SSC have not been documented [16]. The Cochrane review compared two groups (SSC ≤60 minutes and SSC > 60 minutes) and found no evident subgroup differences for the outcomes, including breastfeeding at 1 to 4 months of age, the duration of breastfeeding, infant SCRIP scores at 6 hours, blood glucose, and infant axillary temperature [16]. SSC in isolation may not benefit babies; therefore, future research should investigate the cumulative effects of SSC with other interventions such as early initiation of breastfeeding.

In Cambodia, the practice rate of counselling regarding feeding cues of the babies was 44, and 79% of the babies completed breastfeeding within 90 minutes of birth. As shown in Fig. 4, only 36% of babies completed the first breastfeeding before separation, and the staff tended to provide hands-on breastfeeding support rather than counselling after the babies were returned from undergoing routine care. The study team occasionally observed that hands-on support was provided before the babies showed feeding cues. In contrast, in Lao PDR, the practice rate for counselling was 65, and 54% of the babies completed the first breastfeeding within 90 minutes. Several observational studies showed that approximately 50% of neonates instinctively found the mother’s breast and started suckling at about 1 hour of age when SSC was uninterrupted [21–23]. Data from Lao PDR was comparable with these results. Appropriate support may differ according to different social and cultural backgrounds. The Cochrane review indicated that neonates who are allowed early and uninterrupted SSC and who attach to the nipple on his/her own may continue to nurse more effectively; effective nursing increases milk production and leads to optimal weight gain which contributes to infant survival [16]. The Baby-Friendly Hospital Initiative by UNICEF and the WHO also suggest that health workers should avoid rushing the baby to the breast or pushing the breast into the baby’s mouth [24]. Therefore, hands-on support without considering feeding cues should be kept to a minimum. A study in Turkey showed that breastfeeding support, which included education and training with an infant dummy, provided to mothers of low-birth-weight infants influenced their self-efficacy level, breastfeeding success, and even the growth of the infants [25]. Appropriate breastfeeding support to increase maternal self-efficacy should be pursued according to the situation of the countries.

This study has several limitations. First, the presence of observers could have altered the rates of practices (known as the Hawthorne effect); however, a systematic review about the Hawthorn effect discovered that it is not possible to anticipate which conditions influence the effect, the mechanism of the effect, or the magnitude of the effect [26]. Kurtz et al. measured the Hawthorn effect on hand hygiene practices and found little difference in hand hygiene rates; Leonard et al. found that the effect increases the quality of practices; however, the practices returned to normal after 10 to 15 consultations [27, 28]. The effect may be reduced since our team spent considerable time in each hospital in both countries. Annual reviews in Cambodia and Lao PDR in 2017 showed that the percentage of immediate SSC is 50% in Cambodia and 73% in Lao PDR (national hospitals) in 2015 and 65% in Cambodia (national hospitals) and 52% in Lao PDR (national hospitals) in 2017. The results of the present study demonstrated considerably better rates for these practices [4, 29]. This could be due to the Hawthorn effect; however, it implies that direct observation could increase the awareness of healthcare workers about certain practices. Second, our conclusions may not be applicable to other settings since the health facilities were selected purposefully, and a limited number of deliveries were observed. Lastly, this study did not address the impact of the EENC programme on maternal and infant mortality and morbidity; however, the WHO WPRO and other academic institutions have been collecting information to analyse the impact, and the present study could provide insights into the field regarding the impact of the programme.

Coaching appears to effective in Cambodia and Lao PDR to improve EENC and close the “know-do” gap in both countries. Moreover, these countries showed similar patterns of practice rates throughout the observation periods despite the different situations in each country. Some routine practices appear to be difficult to change, and the quality of the practices could be different even with successful coaching. Several factors may be behind this divergence such as the timing of the study, the perception of the staff or the trainers, and the situation at the health facilities. Interventions to further improve the quality of EENC should be tailor-made according to the situation after careful and regular monitoring.

## Conclusion

The similar practice rates of EENC in Cambodia and Lao PDR suggest that the EENC coaching sessions supported by ministries of health and the WHO WPRO have been effective to close the “know-do” gap. The quality of practices that deviate from the recommendations and practices identified to have lower practice rates should be improved further according to the situation at each country or health facility. Therefore, identifying the quality of practices in a country or health facility through assessment and regular monitoring which are integrated into national health system is important. To further improve the quality of EENC, interventions tailored to each situation are necessary.

## Data Availability

The datasets used and/or analysed during the current study are available from the corresponding author on reasonable request.

## Abbreviations

CI: Confidence interval
EENC: Early essential newborn care
Lao PDR: Lao People’s Democratic Republic
SCRIP: Stability of the cardio-respiratory system
SSC: Skin-to-skin contact
UNICEF: United Nations Children’s Fund
WHO: World Health Organization
WPRO: Regional Office for the Western Pacific

## References

[1] Enweronu-Laryea C, Dickson KE, Moxon SG, Simen-Kapeu A, Nyange C, Niermeyer S (2015). Basic newborn care and neonatal resuscitation: a multi-country analysis of health system bottlenecks and potential solutions. BMC Pregnancy Childbirth.

[2] World Health Organization (2014). Every newborn: an action plan to end preventable deaths.

[3] World Health Organization (2014). Action plan for healthy newborn infants in the Western Pacific region (2014-2020).

[4] World Health Organization (2016). Regional Office for the Western Pacific Region. First biennial progress report: Action plan for healthy newborn infants in the Western Pacific Region (2014-2020).

[5] World Health Organization (2016). Regional Office for the Western Pacific Region. Introducing and sustaining EENC in hospitals: routine childbirth and newborn care.

[6] World Health Organization (2016). Regional Office for the Western Pacific Region.

[7] Spector JM, Agrawal P, Kodkany B, Lipsitz S, Lashoher A, Dziekan G (2012). Improving quality of care for maternal and newborn health: prospective pilot study of the WHO safe childbirth checklist program. PLoS One.

[8] Hirschhorn LR, Semrau K, Kodkany B, Churchill R, Kapoor A, Spector J (2015). Learning before leaping: integration of an adaptive study design process prior to initiation of BetterBirth, a large-scale randomized controlled trial in Uttar Pradesh. India Implement Sci.

[9] Semrau KEA, Hirschhorn LR, Marx Delaney M, Singh VP, Saurastri R, Sharma N (2017). Outcomes of a coaching-based WHO safe childbirth checklist program in India. N Engl J Med.

[10] Semrau KE, Hirschhorn LR, Kodkany B, Spector JM, Tuller DE, King G (2016). Effectiveness of the WHO safe childbirth checklist program in reducing severe maternal, fetal, and newborn harm in Uttar Pradesh, India: study protocol for a matched-pair, cluster-randomized controlled trial. Trials..

[11] Marx Delaney M, Maji P, Kalita T, Kara N, Rana D, Kumar K (2017). Improving adherence to essential birth practices using the WHO safe childbirth checklist with peer coaching: experience from 60 public health facilities in Uttar Pradesh. India Glob Health Sci Pract.

[12] Hirschhorn LR, Krasne M, Maisonneuve J, Kara N, Kalita T, Henrich N (2018). Integration of the opportunity-ability-motivation behavior change framework into a coaching-based WHO safe childbirth checklist program in India. Int J Gynaecol Obstet.

[13] Kara N, Firestone R, Kalita T, Gawande AA, Kumar V, Kodkany B (2017). The BetterBirth program: pursuing effective adoption and sustained use of the WHO safe childbirth checklist through coaching-based implementation in Uttar Pradesh. India Glob Health Sci Pract.

[14] World Health Organization (2009). WHO guidelines on hand hygiene in health care.

[15] World Health Organization (2014). Western Pacific region.

[16] Moore ER, Anderson GC, Bergman N, Dowswell T. Early skin-to-skin contact for mothers and their healthy newborn infants. Cochrane Database Syst Rev. 2016;11:CD003519. 10.1002/14651858.CD003519.pub4 PMC6464366 27885658

[17] Nakamura T, Sano Y (2008). Two cases of infants who needed cardiopulmonary resuscitation during early skin-to-skin contact with mother. J Obstet Gynaecol Res.

[18] World Health Organization (2016). Western Pacific region. Coaching for the first embrace: facilitator's guide.

[19] Bhutta ZA, Das JK, Rizvi A, Gaffey MF, Walker N, Horton S (2013). Evidence-based interventions for improvement of maternal and child nutrition: what can be done and at what cost?. Lancet..

[20] Sobel HL, Silvestre MA, Mantaring JB, Oliveros YE, Nyunt US (2011). Immediate newborn care practices delay thermoregulation and breastfeeding initiation. Acta Paediatr.

[21] Carfoot S, Williamson PR, Dickson R (2003). A systematic review of randomised controlled trials evaluating the effect of mother/baby skin-to-skin care on successful breast feeding. Midwifery..

[22] Righard L, Alade MO (1990). Effect of delivery room routines on success of first breast-feed. Lancet..

[23] Widstrom AM, Lilja G, Aaltomaa-Michalias P, Dahllof A, Lintula M, Nissen E (2011). Newborn behaviour to locate the breast when skin-to-skin: a possible method for enabling early self-regulation. Acta Paediatr.

[24] World Health Organization (2009). Wellstart international baby-friendly hospital intiative: revised, updated and expanded for integrated care. Section 3, breastfeeding promotion and support in a baby-friendly hospital: a 20-hour course for maternity staff.

[25] Kucukoglu S, Celebioglu A (2014). Effect of natural-feeding education on successful exclusive breast-feeding and breast-feeding self-efficacy of low-birth-weight infants. Iran J Pediatr.

[26] McCambridge J, Witton J, Elbourne DR (2014). Systematic review of the Hawthorne effect: new concepts are needed to study research participation effects. J Clin Epidemiol.

[27] Kurtz SL (2017). Measuring and accounting for the Hawthorne effect during a direct overt observational study of intensive care unit nurses. Am J Infect Control.

[28] Leonard K, Masatu MC (2006). Outpatient process quality evaluation and the Hawthorne effect. Soc Sci Med.

[29] World Health Organization (2016). Second biennial progress report: 2016-2017. Action plan for healthy newborn infants in the Western Pacific region: 2014-2020.

